# The global roadmap for advancing development of vaccines against sexually transmitted infections: Update and next steps

**DOI:** 10.1016/j.vaccine.2016.03.111

**Published:** 2016-04-19

**Authors:** Sami L. Gottlieb, Carolyn D. Deal, Birgitte Giersing, Helen Rees, Gail Bolan, Christine Johnston, Peter Timms, Scott D. Gray-Owen, Ann E. Jerse, Caroline E. Cameron, Vasee S. Moorthy, James Kiarie, Nathalie Broutet

**Affiliations:** a World Health Organization, Geneva, Switzerland; b National Institute of Allergy and Infectious Diseases, Bethesda, MD, USA; c Wits Reproductive Health and HIV Institute, University of the Witswatersrand, Johannesburg, South Africa; d Centers for Disease Control and Prevention, Atlanta, GA, USA; e University of Washington, Seattle, WA, USA; f University of Sunshine Coast, Queensland Australia and Institute of Health and Biomedical Innovation, Queensland University of Technology, Brisbane, Australia; g University of Toronto, Toronto, ON, Canada; h Uniformed Services University, Bethesda, MD, USA; i University of Victoria, Victoria, BC, Canada

**Keywords:** Sexually transmitted infections, Vaccines, STI vaccine development, Roadmap

## Abstract

In 2014, the World Health Organization, the US National Institutes of Health, and global technical partners published a comprehensive roadmap for development of new vaccines against sexually transmitted infections (STIs). Since its publication, progress has been made in several roadmap activities: obtaining better epidemiologic data to establish the public health rationale for STI vaccines, modeling the theoretical impact of future vaccines, advancing basic science research, defining preferred product characteristics for first-generation vaccines, and encouraging investment in STI vaccine development. This article reviews these overarching roadmap activities, provides updates on research and development of individual vaccines against herpes simplex virus, *Chlamydia trachomatis, Neisseria gonorrhoeae*, and *Treponema pallidum*, and discusses important next steps to advance the global roadmap for STI vaccine development.

## 1. Introduction

Vaccines against sexually transmitted infections (STIs) are a major priority for sustainable global STI control. Development of new STI vaccines is critical because of the large number of infections worldwide, the resulting adverse sexual, reproductive and maternal-child health outcomes, and important limitations of existing STI interventions. Safe and highly efficacious vaccines against human papillomavirus (HPV) and hepatitis B virus have been major advances in STI prevention and provide inspiration for development of new STI vaccines.

**Fig. 1.**
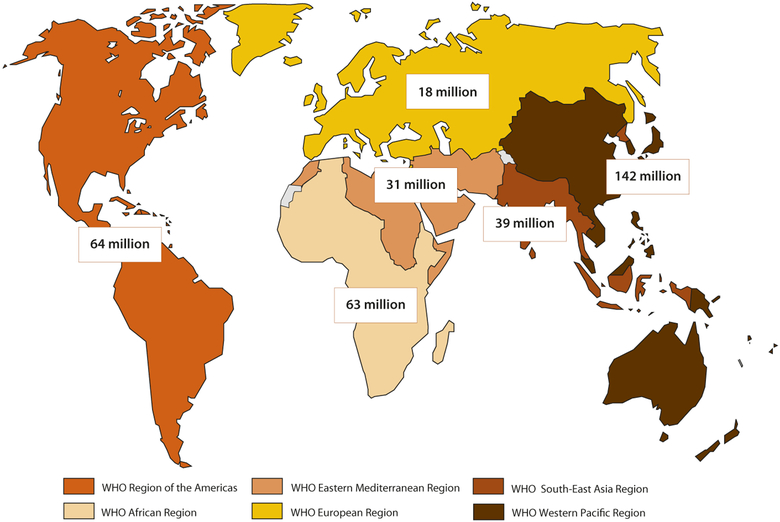
Global and regional estimates of the number of new cases of 4 curable STIs (chlamydia, gonorrhea, syphilis, and trichomoniasis) among 15–49 year-olds in 2012. Global total = 357 million incident infections [1].

**Fig. 2.**
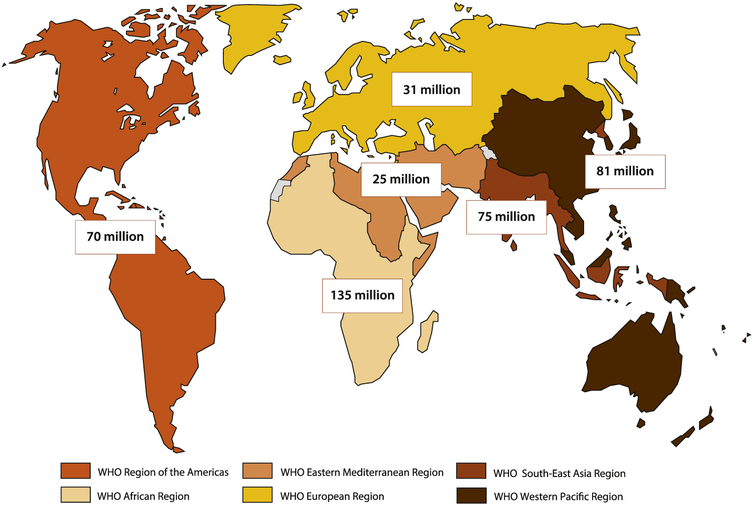
Global and regional estimates of the number of prevalent cases of HSV-2 infection among 15–49 year-olds in 2012. Global total = 417 million prevalent infections [2].

The World Health Organization (WHO) estimates that 357million new cases of curable STIs occurred in 2012 worldwide, including *Chlamydia trachomatis* (chlamydia), *Neisseria gonorrhoeae* (gonorrhea), *Treponema pallidum* (syphilis), and *Trichomonas vagi-nalis* (trichomoniasis) infections [1]. STI numbers were high across all world regions (Fig. 1). In addition, prevalent herpes simplex virus type 2 (HSV-2) infection, the main cause of genital herpes, affected an estimated 417 million people globally in 2012 (Fig. 2) [2], and more than 100 million additional people were estimated to have genital infection withHSV-1 [3].

STIs can result in a number of adverse outcomes. Mother-to-child transmission of syphilis leads to over 300,000 fetal and neonatal deaths per year [4]. HPV causes over 500,000 cases of cervical cancer annually [5]. Chlamydia and gonorrhea are important causes of pelvic inflammatory disease (PID) in women, which can lead to infertility, ectopic pregnancy and chronic pelvic pain [6,7]. In addition, several STIs lead to an increased risk of acquiring or transmitting HIV. HSV-2 infection increases the risk of HIV acquisition by three-fold [8]. Not to be forgotten, the genital symptoms and psychosocial consequences of STIs have important effects on quality of life.

Current STI control is challenged by several factors [9]. First, although condoms are an important STI prevention tool, there have been limits to progress made with condoms as the main primary prevention measure. Second, most STIs are asymptomatic, and availability of affordable, feasible and rapid tests is lacking in many settings. Especially in lower-income countries, most infections are not diagnosed. Chlamydia screening programs for young women have been difficult to bring to scale in high-income countries and by themselves, without a strong focus on partner treatment, have not resulted in clear reductions in sexual transmission of chlamydia infections [10]. Third, the growing threat of antimicrobial resistance, with increasing reports of cephalosporin-resistant gonorrhea, creates an urgent need for new prevention tools [11]. Supply chain shortages of antibiotics, e.g., benzathine penicillin for syphilis, are also a major concern. Finally, STIs are often stigmatizing and have received little public policy attention. Without a simple, clearly effective intervention, it has been difficult to garner support [9,12]. Thus, while efforts to scale up existing interventions continue, these challenges highlight the need for ongoing work toward STI vaccine development.

## 2. STI vaccine roadmap

In 2013, WHO and the National Institutes of Health (NIH) organized a technical consultation to evaluate how to advance STI vaccine development. The consultation resulted in a special issue of the journal *Vaccine* in 2014, which included articles on the prospects for new vaccines against HSV, chlamydia, gonorrhea, syphilis, and trichomoniasis [13]. The special issue concluded with a proposed global roadmap for STI vaccine development [14]. The roadmap was developed by consensus, based on discussions at the technical consultation, and outlines critical next steps from prevaccine development through vaccine introduction.

Vaccine development is a long, expensive and risky process, which progresses along a defined development pathway [15]. In the discovery stage, basic science research aims to understand the disease and protective immune mechanisms in order to select a candidate vaccine. Typically, preclinical studies in animal models evaluate these candidates. Clinical development involves human studies. Small Phase I studies evaluate safety, while slightly larger Phase II trials further characterize safety, immunogenicity, formulations and doses. Phase III studies evaluate vaccine efficacy and safety in large randomized, placebo-controlled trials. Successful vaccines then need regulatory approval. At each stage, several “go”/“no go” decisions determine whether the process moves forward, particularly in early development as a candidate is optimized before commitment to expensive Phase III trials. “Push” forces, such as advances in technology or an influx of funding, and “pull” forces, such as a clearly defined disease burden or strong market for the vaccine, can help accelerate vaccine development through the stages.

The STI vaccine roadmap outlines 9 priority action areas, with specific action steps, that can generate push and pull forces and catalyze steps in between, to advance vaccine development (Table 1). This article presents an overview of key overarching activities undertaken to date to advance the STI vaccine roadmap in the following areas: obtaining better epidemiologic data, modeling the impact of STI vaccines, advancing basic science research, defining preferred product characteristics, and encouraging investment in STI vaccine development. We then provide updates on development of individual STI vaccines.

## 3. Overarching advances in implementing the STI vaccine roadmap

### 3.1. Obtaining better epidemiologic data on STI burden

One of the key action items in this area of the roadmap was to update global and regional estimates of STI burden. In 2015, WHO updated estimates of the global prevalence and incidence of HSV-2 infection [2], and released the first-ever global estimates of HSV-1 infection [3]. An estimated 417 million people aged 15–49 years had HSV-2 infection (11.3% prevalence) in 2012 [2]. HSV-1 infection affected an estimated 3.7 billion people aged 0–49 years (67% prevalence), of whom an estimated 140 million people had HSV-1 genital infection [3]. Thus, more than 500 million people aged 15–49 years had genital infection with either HSV-1 or HSV-2 [2,3]. These figures are currently being used to generate estimates of the burden of neonatal herpes infections, expected in 2016.

HSV-2 infection was also added to conditions evaluated in the Global Burden of Disease (GBD) Study 2013, which estimated that HSV-2 infection resulted in 311,600 years lived with disability (YLD) in 2013 from genital ulcer disease alone [16]. These YLD estimates do not include disability due to neonatal herpes nor HSV-associated HIV acquisition, which are the most devastating consequences of infection. The GBD study has ongoing updates, which will continue to refine the methodology for evaluating YLD associated with HSV, including consideration of adding HSV as a risk factor for HIV.

Updated curable STI estimates were also published in 2015[1]. In 2012, there were an estimated 131 million new chlamydia infections, 78 million new gonorrhea infections, 5.6 million new syphilis infections and 143 million new trichomoniasis infections[1]. The GBD 2013 study demonstrated that although syphilis had the fewest number of cases, its associated fetal and neonatal complications meant syphilis had the highest disability-adjusted life years lost of all the curable STIs [17]. In many regions, and especially for men, these STI estimates were constrained by a paucity of published prevalence data. Following a consultation meeting in 2015, WHO is pursuing strategies to strengthen country-level STI surveillance and improve the methodology for future estimates. Improved global data on the burden of PID and infertility related to chlamydia and gonorrhea will also be important.

As de novo dedicated STI burden studies may be too resource-intensive and not realistic in many settings, another roadmap recommendation was to partner with research sites to leverage existing but non-published STI data. One such project that has emerged from these discussions is STIMA, an STI individual participant data meta-analysis using combined data from 18 prospective HIV prevention studies, which were initially combined to look at the effect of hormonal contraception on HIV acquisition [18]. These data represent over 37,000 women in sub-Saharan Africa, more than 95% of whom have data with which STI prevalence or incidence can be assessed. In the future, in addition to newer HIV prevention trials, it will be important to consider evaluating STI data from HPV vaccine, circumcision, and contraception trials. It will also be important to continue to seek opportunities to add STI variables to surveillance studies and clinical and prevention trials, especially from the outset.

### 3.2. Modeling the theoretical impact of STI vaccines

An HSV vaccine modeling meeting was held at WHO in March 2015 to review existing models and identify modeling needs related to HSV infection, costs and the theoretical impact of an HSV vaccine. Several existing models showed that even an imperfect HSV vaccine could be beneficial [19–21]. However, the group outlined several new modeling needs critical to advancing HSV vaccine development and decision-making in the future, such as inclusion of HSV-associated HIV incidence and neonatal herpes as model outcomes and incorporation of HSV-1 infection, both as an outcome and as a potential modifier of the impact on HSV-2. The group also recommended modeling in different epidemiologic and economic settings, and inclusion of cost-effectiveness analyses. In order to conduct these models, better primary data from low- and middle-income countries (LMICs) on neonatal herpes and on the costs of HSV will be crucial. The planned Child Health and Mortality Prevention Surveillance (CHAMPS) network [22], which will explore the causes of neonatal deaths in developing countries, may allow collection of much needed data on fatal neonatal HSV infections.

New chlamydia vaccine modeling efforts in 2015 include work suggesting that a successful chlamydia vaccine could be cost-effective [23]. Although there are many unknowns related to chlamydial vaccine development, these kinds of exercises lay the framework for thinking through pertinent assumptions and model structure, and can be updated as vaccine development advances.

### 3.3. Advancing basic science research

The US National Institute of Allergy and Infectious Diseases (NIAID), part of the NIH, has held several workshops focused on advancing basic science for STI vaccine development. The report of a workshop for HSV vaccine development was published in the special issue of the journal *Vaccine* in 2014 [24]. Workshops were also held to focus on gaps and challenges for the development of chlamydia and gonorrhea vaccines. In May 2015, chlamydia researchers from around the world gathered to discuss the status of chlamydia vaccine development, gaps in current knowledge of immune responses and in correlates of protective immunity, the potential benefits of an effective chlamydia vaccine, and issues pertaining to clinical testing of vaccine candidates. A similar meeting was held in June 2015 to discuss a vaccine to prevent gonococcal infection. Reports from both workshops will be released in 2016.

One of the goals of these workshops was to identify potential reagents, immunogens, and assays that, if standardized and made available to researchers, might accelerate moving vaccine candidates from animal studies into clinical evaluation. To facilitate this process, NIAID offers targeted product development support to assist in identifying and filling critical gaps in the product development pipeline.

### 3.4. Defining preferred product characteristics

Preferred product characteristics (PPCs) reflect formal WHO guidance on the desired parameters of a vaccine that would meet priority public health goals, especially for LMICs [25]. A PPC document describes characteristics such as vaccine indications, target groups, possible immunization strategies, and desired clinical trial data related to safety and efficacy, e.g., whether the endpoint is prevention of morbidity or infection, or the efficacy requirements to achieve a minimum public health effect [26]. PPCs are intended to provide early guidance to any entity intending to eventually seek WHO vaccine prequalification and policy recommendations, and may be distinguished from industry-generated target product profiles in that they are tailored specifically to the needs of LMICs. PPCs are derived through a stakeholder consultation process that establishes a shared global vision and are generated for vaccines that WHO considers a public health priority. Thus, they engage regulators and policy-makers early on and can catalyze industry support and shape target product profiles used by industry and by some funding entities.

The Product Development for Vaccines Advisory Committee (PD-VAC) provides strategic advice to WHO related to vaccines in early clinical development, and makes recommendations related to developing PPCs for a particular vaccine. HSV vaccines were reviewed at the 2015 PD-VAC meeting (background paper by Johnston et al. [27]). Because of the global immunization focus on preventing deaths in children under 5 years, PD-VAC recommended studies to obtain better data on neonatal herpes in LMICs, in particular through the CHAMPS evaluation. Because HSV-2 infection is associated with an increased risk of HIV infection, with an estimated population attributable risk percentage of 48% in some populations [28], the committee encouraged modeling studies to evaluate the potential for HSV vaccines to reduce HIV infection rates. Finally, PD-VAC supported the development of a robust investment case, including assessment of potential vaccine impact on a broad array of HSV-2 and HSV-1 outcomes, in order to catalyze further HSV vaccine research and development.

### 3.5. Encouraging investment in STI vaccine development

**Fig. 3.**
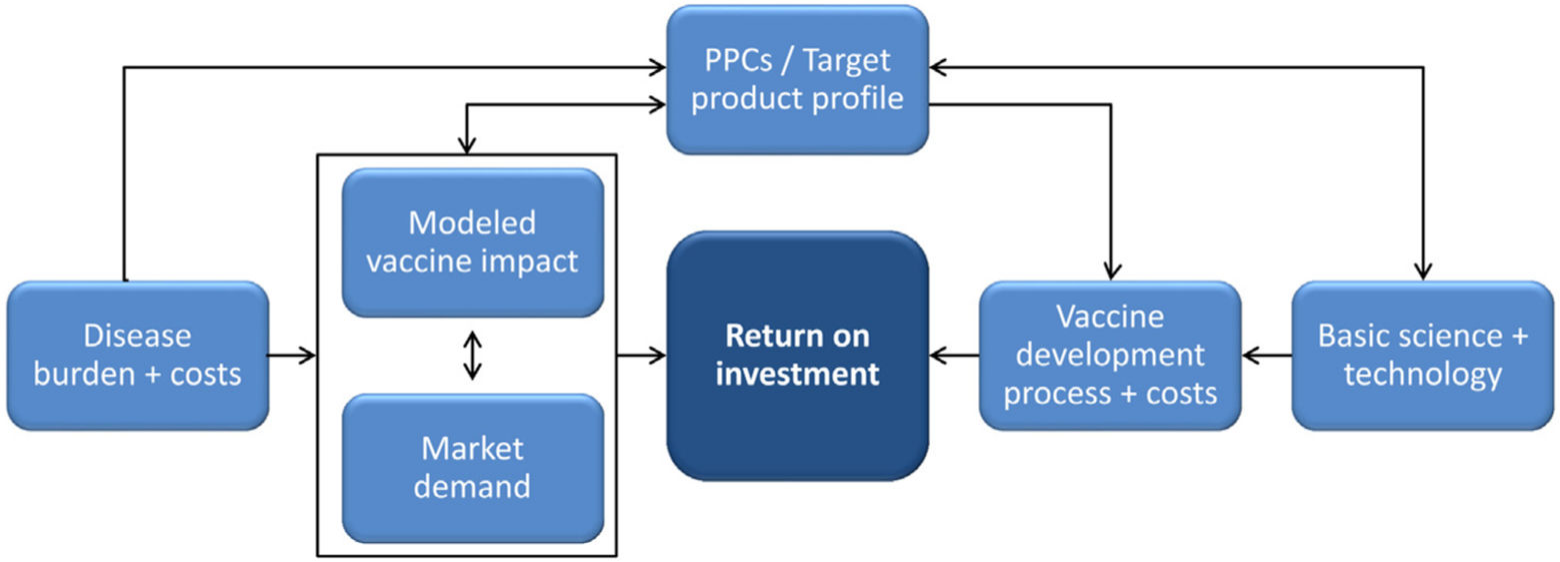
Elements of the comprehensive vaccine business case. PPCs = preferred product characteristics.

In line with the STI vaccine roadmap and PD-VAC recommendations, WHO aims to generate a comprehensive business case for HSV vaccine development to outline the public health rationale for the vaccine. The business case first demonstrates the public health need for the vaccine in terms of the disease burden and costs (Fig. 3). This helps determine the market for the vaccine, within the context of competing interventions, and allows modeling of potential vaccine impact and cost-effectiveness. Outlining disease burden epidemiology and modeling vaccine impact also facilitate defining preferred product characteristics. These PPCs, along with basic science and technology, determine the precise vaccine development pathway and costs. The balance of the projected market demand and vaccine impact with the projected costs of vaccine development determines the potential return on investment. Once available, the business case document will help inform decision-making and rationalize investment by varied stakeholders, including donors, vaccine developers and manufacturers, and policymakers. To date, a detailed work plan and budget for an HSV vaccine business case has been developed. Business cases for chlamydia, followed by other STI vaccines, will also be instructive as development of these vaccines advances.

Raising awareness about STI vaccine development is also critical for encouraging investment. Since development of the STI vaccine roadmap, the global introduction of HPV vaccine, including in LMICs, has produced widespread recognition that STI vaccines can be successfully introduced. The global need for development of STI vaccines has also been highlighted in a number of academic forums, such as the Global Vaccine and Immunisation Research Forum and the World STI and HIV Congress.

## 4. Progress in research and development of individual STI vaccines

**Fig. 4.**
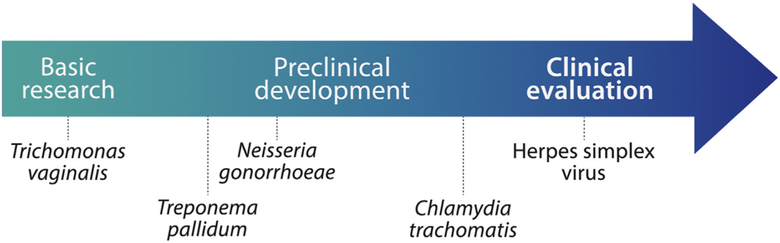
Current status of the development pathway of STI vaccines.

The current status of the development pathway for STI vaccines is shown in Fig. 4. Vaccine development efforts are furthest along for HSV, for which several vaccine candidates are in Phase I and II trials. Progress has been made in the preclinical development of chlamydia vaccine candidates, with the first Phase I clinical trials expected to start in 2016. Vaccine development is not as advanced along the development pathway for gonorrhea and syphilis, but candidates are emerging that could be developed over the next several years. Updates on the progress in research and development for these four vaccines follow. The path to vaccine development for trichomoniasis is the least understood, with more basic science research needed, as well as better epidemiologic and natural history data.

### 4.1. Herpes simplex virus

The STI vaccine roadmap catalyzed the HSV vaccine field in several important ways. In addition to the new global estimates of HSV-1 and HSV-2 infection demonstrating the high burden of HSV infection worldwide, the roadmap also stimulated discussion about the PPCs of an HSV vaccine. A prophylactic vaccine to prevent genital HSV infection, which would be effective in both HSV-1 seropositive and seronegative persons and could be given either as part of the adolescent or childhood platform, would be ideal. The most recent prophylactic Phase III HSV vaccine trial tested a subunit glycoprotein D2 vaccine to prevent symptomatic genital herpes disease in 8323 North American HSV-1/HSV-2 seronegative women[29]. The vaccine demonstrated efficacy of only 20% against genital herpes disease, which did not justify continued development of this candidate. However, several important findings emerged from this landmark trial. Surprisingly, the overall attack rate for symptomatic genital HSV disease was only 1.1% [29], suggesting that future trials should consider either an alternative endpoint, such as HSV infection, or be conducted in individuals at greater risk of genital HSV disease. Furthermore, of the 183 women in the trial who became infected with HSV, only 62 (34%) had symptomatic or suspected genital disease and 121 (66%) acquired HSV-1, demonstrating the importance of both asymptomatic infection and the emerging role of genital HSV-1 [30]. Finally, higher antibody titers to gD-2 were associated with increased vaccine efficacy against HSV-1 infection and disease, suggesting the first immune correlate of protection against HSV [31]. The results from this trial will be instrumental in defining PPCs and designing future prophylactic HSV vaccine trials.

Despite the disappointing efficacy results of the Phase III trial, the HSV vaccine field remains very active. Five vaccine candidates have entered Phase I/II testing over the past two years, and numerous other candidates are in development. These vaccine candidates are reviewed in more detail in the HSV vaccine development brief by Johnston et al. [27]. Four vaccine candidates being evaluated in early clinical studies use novel epitope/adjuvant combinations, and one candidate in Phase I studies, HSV529, is a live, replication-defective HSV-2 vaccine candidate with deletions in UL5 and UL29[32]. Most of the vaccines in clinical trials are currently being tested in persons with HSV-2 infection to reduce genital herpes recurrences and shedding (“therapeutic vaccination”) rather than to prevent infection among HSV seronegative individuals. These early phase studies of therapeutic HSV vaccines are using a reduction in the frequency of genital HSV shedding as the primary clinical endpoint, rather than time to first genital herpes recurrence, which has been used in previous studies [33]. Because HSV shedding is in the causal pathway for HSV recurrences, it is an excellent biomarker for HSV disease severity [34].

Promising preliminary results from the Phase II trial of the GEN-003 therapeutic vaccine, a protein subunit vaccine with gD2 and ICP4 and Matrix M2 adjuvant, have shown an approximate 50% decline in the genital HSV shedding rate, with similar interim results seen in a dose-finding Phase II trial [35]. A Phase II study of the HerpV vaccine, a 32-peptide vaccine linked to heat shock protein and QS-21 adjuvant, showed a 15% decline in HSV shedding frequency. Results from two DNA vaccines currently in Phase II trials are eagerly awaited [36,37]. Other advances in the field include the increasing number of full-length HSV genomes which have been sequenced from around the world [38–40]. Sequencing data have revealed that vaccine candidate glycoprotein D is highly conserved between HSV-1 and HSV-2 [41]. Data showing that gD and gB stimulate the dominant neutralizing antibody response to HSV, as well as greater understanding of the importance of type-specific neutralizing antibody responses to HSV infection will guide vaccine development [42]. In addition, recently identified tissue resident memory CD8+ T-cells may be important to stimulate, for both prophylactic and therapeutic vaccination [43]. The recent momentum in the HSV vaccine field lays the groundwork for novel approaches to HSV vaccine design and provides hope that effective HSV vaccines may be developed in the near future.

### 4.2. C. trachomatis

The prospects for a *C. trachomatis* vaccine are increasingly promising, primarily because the last 3 years has seen the rapid development of new tools for *Chlamydia* research that will accelerate vaccine development. One of the major developments has been the long-awaited technology to genetically manipulate *Chlamydia*[44]. By paying careful attention to detail, it is possible to cure *C. trachomatis* of its native plasmid and then to engineer the plasmid to contain an *Escherichia coli* origin and subsequently re-introduce the plasmid with a testable foreign gene. An important complementing strategy has been the production of mutant libraries. At this time, these libraries are not usually isogenic (i.e., only contain changes in a single gene). Nevertheless, because it is relatively inexpensive to sequence cloned isolates from such libraries, and these cloned mutant strains can be tested both in vitro and in vivo, several groups are now making use of this technology to identify and characterize novel virulence genes [45–48]. One laboratory has tested each of the open reading frames of the chlamydial plasmid and has identified key roles for pgp3 and pgp4 [45,49]. Another has used this strategy to analyze an increasing number of key chlamydial proteins [46–48]. Together, these approaches are facilitating identification and validation of novel vaccine targets.

While the *Chlamydia muridarum* mouse model has long been used by chlamydiologists for both basic science and vaccine development, it is widely acknowledged to have several shortcomings. Work has therefore continued to utilize the non-human primate model and this has enabled testing of an attenuated plasmid-free chlamydial strain as a potential trachoma vaccine[50]. Overall the results are quite promising, with the plasmid-free strain of C. trachomatis producing very mild eye infections and providing significant protection against wild type challenge. There is a word of warning as subtle differences in the ocular versus genital tract sites may result in unexpectedly different outcomes [51]. A novel mini-pig model has allowed evaluation of a multi-subunit major outer membrane protein (MOMP)-derived vaccine candidate against sexually transmitted chlamydial infections, which was highly immunogenic and offered promising levels of protection against vaginal *C. trachomatis* infection in immunized pigs [52]. A first-generation vaccine candidate based on this technology is scheduled to enter phase I clinical trials in 2016 [53].

A key aspect of any vaccine work is to develop correlates of protection and to better understand the mechanisms of immunity. A key publication in 2015 showed that by utilizing novel adjuvant-antigen strategies (charge-switching synthetic adjuvant particles), it was possible, using the mouse model, to not only stimulate a strong CD4 response to chlamydial infection, but also to direct it to the genital mucosa where it is required [54]. For a genital *C. trachomatis* vaccine to be effective, it will likely need to elicit tissue-resident T cells. While the chlamydial MOMP has long been the focus of vaccine development, several new candidate antigens (e.g., polymorphic membrane proteins [PMPs]) are emerging and are showing great promise, in both mouse and primate models. Combined with the live attenuated vaccine strategy, plus an expanding list of novel adjuvants, there is great promise for more vaccine candidates to be developed. Overall, new tools and new discoveries certainly bring new hope to *C. trachomatis* STI vaccine development, and it is likely that several candidate vaccines will enter Phase I clinical trials in the next few years.

### 4.3. N. gonorrhoeae

Despite the intense innate inflammatory response that is the hallmark of *N. gonorrhoeae*, there is no naturally acquired immunity to the bacteria, making it difficult to predict which type(s) of response might be protective. Moreover, *N. gonorrhoeae* is exquisitely adapted to life in humans, and this host restriction has hampered efforts to model infection or disease, which would provide insight as to how these bacteria persist within the infected mucosa. While these challenges have hampered progress on vaccine development, the urgency associated with emergence of multidrug-resistant gonococcal strains has dovetailed with recent advances in genomic diversity, modeling infection and our understanding of immunity to re-invigorate the field.

Perhaps the single greatest hurdle in the development of a gonococcal vaccine stems from the host restriction of these bacteria, which makes it difficult to establish gonococcal infection in laboratory animals. Demonstration that estradiol-treated mice become colonized with *N. gonorrhoeae* after vaginal inoculation was a major advance in this regard, providing a model in which candidate vaccines can be tested [55]. Studies using this model have recently provided new insight into the immune response to infection. Specifically, normal infection elicits an inflammatory Th17 response that precludes development of adaptive immunity; if cytokine or other adjuvants are used to skew this to a Th1-based response, mice become immune to repeat infection [56,57]. The presence of host restrictions also makes it difficult to use animal models to appreciate the contribution of virulence factors that are specific for humans. Ongoing work to ‘humanize’ mice, such as by generation of transgenic animals expressing human CEACAM receptors to which gonococci adhere, human transferrin and lacto ferrin that they use for iron, and human-derived complement regulatory proteins that gonococci use to evade complement-mediated killing, has provided models of uncomplicated lower genital tract infection and pelvic inflammatory disease that more closely reflect gonococcal infection within the human genital mucosa. These provide an exciting platform to focus vaccine development efforts on targets that provide essential functions, and to test the potential impact of antigen-specific immune responses against these targets.

While more sophisticated mouse models allow systematic evaluation of vaccine antigens, adjuvants and immunization routes, and enable a search for correlates of protection, it is clear that some aspects of immunity may differ between mice and humans. This has motivated efforts to re-establish clinical cohorts to study the natural history of gonococcal infection [58], provide samples with which to understand genome [59] and transcriptome [60] diversity among gonococcal isolates, and to exploit ‘omics’-level approaches to discriminate between protective and pathogenic host responses. These studies will undoubtedly provide a richer understanding of the gonococcal lifestyle and help validate various aspects of the mouse-based studies. Providing a critical link between these two approaches has been the recent re-establishment of experimental human male urethra infection studies at the University of North Carolina at Chapel Hill [61]. While restricted in its capacity to assess large numbers of variables, the importance of this model for confirming the contribution of virulence factors or establishing the efficacy of candidate vaccines cannot be overstated.

The emergence of multidrug-resistant *N. gonorrhoeae* has led to a global awareness of the urgent threat presented by this pathogen. This has prompted genome sequencing-based efforts to understand drug resistance, which provides a population-level view of gonococcal genetic diversity that allows the variability of putative vaccine targets to be appreciated. This new understanding is emerging in parallel with the sophistication of preclinical mouse, experimental human and population-based studies of gonococcal infection. Momentum in each of these endeavors has led gonococcal investigators to aim to integrate the varied efforts being undertaken to develop new vaccines, and to develop a pipeline of candidates to expedite the path into clinical trials and support the goal of delivering an effective gonococcal vaccine.

### 4.4. T. pallidum

Although some progress has been made in reducing the number of syphilis infections globally [1], syphilis remains an important cause of fetal and neonatal mortality in many LMICs [4,62], and outbreaks have continued to occur in several high-income countries [63,64], especially among gay, bisexual and other men who have sex with men who are often co-infected with HIV [65]. The ongoing threat posed by syphilis highlights the crucial need for continuing work to develop an effective syphilis vaccine. However, only a limited number of investigators worldwide work on syphilis vaccine development. One body of research primarily focuses upon deciphering the complexities associated with the process of antigenic variation within *T. pallidum* and establishment of persistent and recurrent infection [66–69]. Another focuses upon treponemal dissemination within the host [70–73]. The goal of both areas of research is to use a reverse vaccinology approach, combined with targeted functional studies, to fully understand critical host–pathogen interactions and key pathogenic mechanisms utilized by *T. pallidum*, at least some of which appear to be novel and exquisitely tailored for the requirements of an obligate pathogen. Information from these studies is then used to identify optimal syphilis vaccine targets.

The major advance in syphilis vaccine development over the last two years has been the commitment by investigators to join forces and propose a two-pronged approach to prevention of syphilis infection, whereby a vaccine cocktail of antigens critical for chancre development [66–69] are combined with an antigen essential for treponemal dissemination [70–72]. Such an approach, if successful, would deliver a “holistic” vaccine that stems transmission of syphilis during the primary stage of infection via prevention of ulcerative chancres, and simultaneously prevents the serious sequelae (congenital infection, neurosyphilis) and secondary stage transmission that occur due to treponemal dissemination.

A path moving forward for syphilis vaccine development is vital to evaluate the currently available vaccine approach and to attract new investigators to the field to develop additional vaccine targets and strategies. Advances in sequencing of circulating syphilis strains can provide additional information on the cross-protective potential of selected vaccine targets [74,75]. Ongoing studies investigating immune correlates associated with protection from disease, adjuvant optimization to achieve these immune functions, and mathematical modeling studies to predict the global health impact of a syphilis vaccine will also be valuable. Widespread support and innovative partnerships will be needed to catalyze syphilis vaccine development and combat a global disease that has devastating consequences for reproductive and fetal/newborn health, affecting the general population of many LMICs and key populations worldwide.

## 5. Moving forward

The STI vaccine roadmap outlines priority action steps to advance STI vaccine development. Since its publication, much progress has been made in several overarching activities to obtain better epidemiologic data to establish the public health rationale and global market for these vaccines, to model the theoretical impact of future vaccines, and to establish what is needed to define preferred product characteristics and encourage investment in STI vaccine development. Several basic science advances for individual vaccines have benefited from parallel efforts to coordinate and streamline research and product development among investigators. The greatest advances have been made with HSV vaccine development, where multiple promising vaccine candidates in early clinical trials provide hope that an HSV vaccine is on the horizon. However, the first new sexually transmitted chlamydia vaccine candidate will be entering phase I trials, and new technology advances will see several more vaccine candidates in the pipeline.

A confluence of factors, such as international roll-out of HPV vaccine raising global awareness of a successful STI vaccine, the growing specter of multidrug-resistant gonorrhea, technological advances in antigen selection and novel adjuvants, and the STI vaccine roadmap itself, has provided renewed enthusiasm for STI vaccine development. Interest in STI vaccine development has dovetailed with the growing movement around development of multipurpose prevention technologies, which combine various HIV, STI and pregnancy prevention features [76]. Both efforts heighten awareness of the importance of harnessing new technology advances to improve sexual and reproductive health worldwide. In order to capitalize on this momentum, the goal is to expand on the substantial collaborative efforts already undertaken by the STI vaccine community to develop an overarching STI vaccine consortium, with working groups for each STI. The STI vaccine roadmap provides a framework to coordinate and streamline activities so that they contribute to a larger cohesive whole. HSV vaccine has been prioritized in initial efforts, in terms of conducting impact modeling, generating PPCs, and developing a business case, as its research and development program is furthest along. These types of activities are now needed for chlamydia and gonorrhea vaccines as well, along with redoubled efforts to jumpstart syphilis vaccine development. With continued support and collaboration, these much needed STI vaccines can be made a reality.

## Figures and Tables

**Table 1:** STI vaccine roadmap: nine priority action areas.

1. Obtain better epidemiologic data on STI burden	6. Define preferred product characteristics for 1st generation vaccines
2. Improve understanding of STI natural history and sequelae	7. Expedite clinical development and evaluation
3. Model the theoretical impact and cost-effectiveness of STI vaccines	8. Plan for vaccine introduction in advance
4. Advance basic science research for STI vaccines	9. Encourage investment in STI vaccine development
5. Conduct basic and translational studies in human clinical settings as soon as possible	
